# The melanocyte lineage in development and disease

**DOI:** 10.1242/dev.106567

**Published:** 2015-02-15

**Authors:** Richard L. Mort, Ian J. Jackson, E. Elizabeth Patton

**Affiliations:** 1 MRC Human Genetics Unit and; 2 University of Edinburgh Cancer Research UK Cancer Centre, MRC Institute for Genetics and Molecular Medicine, University of Edinburgh, Edinburgh EH4 2XU, UK; 3 Roslin Institute, University of Edinburgh, Edinburgh EH25 9RG, UK

**Keywords:** MITF, Melanoma, Neural crest, Stem cells

## Abstract

Melanocyte development provides an excellent model for studying more complex developmental processes. Melanocytes have an apparently simple aetiology, differentiating from the neural crest and migrating through the developing embryo to specific locations within the skin and hair follicles, and to other sites in the body. The study of pigmentation mutations in the mouse provided the initial key to identifying the genes and proteins involved in melanocyte development. In addition, work on chicken has provided important embryological and molecular insights, whereas studies in zebrafish have allowed live imaging as well as genetic and transgenic approaches. This cross-species approach is powerful and, as we review here, has resulted in a detailed understanding of melanocyte development and differentiation, melanocyte stem cells and the role of the melanocyte lineage in diseases such as melanoma.

## Abstract (precis)

**Summary**: This Review discusses melanocyte development and differentiation, melanocyte stem cells, and the role of the melanocyte lineage in diseases such as melanoma.

## Introduction

Melanin, the pigment that colours the skin, eyes, hair, fur, feathers and scales of vertebrates (see Box 1), is made by specialised cells called melanocytes. Some animals, such as birds, fish and reptiles, have additional cells that make other pigments that, through reflection and refraction, display a wide variety of colours, but melanocytes are ubiquitous across vertebrates. Studies of melanocyte biology played a major role in the birth of genetics. In the first genetic experiments in animals, for example, Cuenot (1902) used crosses of unpigmented albino mice to confirm Mendel's Laws. Genetics has continued to play an important role in aiding our understanding of melanocyte biology. Most notably, there are almost 200 genes identified in mice that play a role in pigment biology, and numerous genes in other species, in particular zebrafish, that together govern melanocyte development and cell biology.

Box 1. **Melanosomes and melanin production**

Melanin is a macromolecule synthesised by melanocytes. The first step in melanin synthesis is catalysed by the enzyme tyrosinase, which converts tyrosine to dihydroxyphenylalanine (DOPA). The lack of this enzyme results in albinism. Melanin in mammals and birds comes in two forms: eumelanin, which is black or dark brown, and phaeomelanin, which is red or yellow. The biosynthetic pathways for the two pigments diverge downstream of DOPA, and the choice of pathway is determined by the signalling activity of the melanocortin receptor MC1R. The biosynthetic intermediates of melanogenesis are toxic, which is perhaps one reason why the synthesis occurs in a specialised organelle, a modified lysosome known as melanosome. Most melanocytes do not retain melanin, but rather the melanin granules are transported along microtubules to the tips of dendrites, long cell protrusions, from where they are transferred to nearby keratinocytes in the skin, hair or feather. The situation in fish is somewhat different: melanocytes only produce eumelanin and the melanin granules are retained by the cell. MC1R activity in fish produces a movement of the melanin granules out along microtubules, which results in a darkening of the fish as camouflage. The reverse movement of melanin into the centre of the fish melanocyte is produced via a different signalling system involving the melanin-concentrating hormone (MCH) receptor.

Melanocytes and their progenitor cells, melanoblasts, have also long been of particular interest to developmental biologists. They provide an excellent model for development: they are a single cell type that differentiates from a multipotential stem cell, they migrate through the developing embryo and interact with their environment to localise to specific sites in the body, and they generate a stem cell population through which they self-renew. These properties might be one of the reasons that melanoma, the cancer originating from melanocytes, is particularly aggressive and metastatic. In this Review, we bring together recent work from mice, birds and zebrafish that together provide a powerful cross-species perspective on melanocyte development and cancer biology. We review the transcriptional regulation of the development of melanoblasts and melanocytes, and how their migration is controlled. Furthermore, we review recent advances made in understanding the biology of melanocyte stem cells (MSCs), which aid our understanding of the biology of stem cells in general. Finally, we discuss how our knowledge of melanocyte biology has informed research on melanoma and is contributing to the design of new treatment strategies.

## The neural crest and the origins of the melanocyte lineage

The melanocyte lineage is derived from the neural crest, which has its origins in the neural tube. Following its formation, neural crest cells delaminate from the dorsal-most aspect of the neural tube by a process of epithelial-to-mesenchymal transition. These neural crest cells are highly migratory and go on to form many specialised structures and tissues in the developing embryo by migration, proliferation and differentiation (Mayor and Theveneau, 2013). The antero-posterior position at which neural crest cells delaminate broadly defines their fate and, based on this positioning, the neural crest can be subdivided into five overlapping groups: cranial, vagal, sacral, trunk and cardiac. The neural crest in the trunk region gives rise to, among others, melanocytes, neurons and glia. Elegant work using avian grafts demonstrated that cells that leave the crest early follow a ventral migration path through the anterior sclerotome and become neurons and glia, whilst late departing cells follow a dorsolateral pathway between the ectoderm and the somites through the developing dermis and become melanocytes (Erickson and Goins, 1995). The fate of the cells is not determined by their migratory path, but rather the cells are specified before leaving the crest. By isolating single migratory neural crest cells and allowing their differentiation *in vitro*, it was shown that, whilst some isolated cells could give rise to mixed clones containing neurons, glia and melanocytes, and others were fated to produce only a single cell type, two other major mixed-fate cells were identified: some cells were progenitors of glia and neurons, whilst others could produce glia and melanocytes (Henion and Weston, 1997). Furthermore, Schwann cells, which are the glial cells of the peripheral nervous system, can be dedifferentiated *in vitro* to a glia/melanocyte precursor (Dupin et al., 2003). The fate map is thus slightly complicated, with glia seeming to have two different origins. Recent work, discussed in detail below, also suggests a dual neural crest origin of melanocytes, one by early differentiation from the neural crest and dorsolateral migration, and the other from a Schwann cell (glial)/melanoblast progenitor on the ventral path.

## Transcriptional regulation of melanocyte identity

**Fig. 1.**
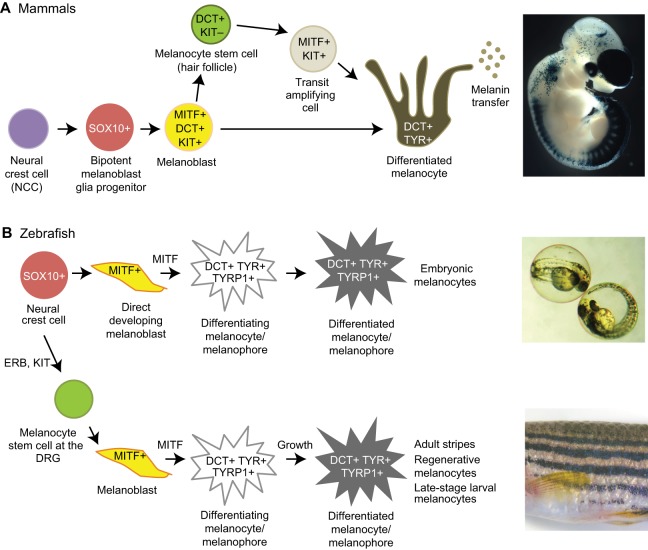
**An overview of melanocyte development.** (A) In mammals, melanoblasts are specified from neural crest cells (NCCs) via a SOX10-positive melanoblast/glial bipotent progenitor. SOX10 expression remains switched on in both of these lineages. Melanoblasts subsequently are specified and acquire MITF, DCT and KIT expression. After colonising the developing embryonic hair follicles, some melanoblasts differentiate into melanocytes and produce the pigment (melanin) that colours the first hair cycle. A subset of melanoblasts dedifferentiate (losing MITF and KIT expression but not DCT) to form melanocyte stem cells in the hair follicle bulge that replenish the differentiated melanocytes via a rapidly proliferating transit-amplifying cell in the subsequent hair cycles. The image on the far right is of a transgenic mouse embryo expressing *lacZ* under control of the melanoblast promoter *Dct*. X-Gal staining reveals blue-stained melanoblasts, in particular those migrating from the cervical neural crest and in the head. Also stained are the telencephalon, the dorsal root ganglia (DRG) and the retinal pigmented epithelium of the eye. (B) In zebrafish, there are distinct embryonic and adult pigmentation patterns, as illustrated in the images on the far right. The melanoblasts that form both these patterns originate from a SOX10-positive neural crest-derived progenitor. The embryonic pattern is formed by melanocytes that develop directly from this progenitor via an MITF+ melanoblast. The melanoblasts that form the adult pattern are derived from a melanocyte stem cell population that resides at the dorsal route ganglia (DRG) and is specified by an ERB- and KIT-dependent pathway in the embryo.

The transcription factor microphthalmia-associated transcription factor (MITF) appears to be the master regulator of melanocyte identity and is embedded within a transcriptional network (Fig. 1) that controls the development of melanocytes from the neural crest [reviewed by Baxter et al. (2010)]. Mice lacking MITF cannot form melanocytes (Steingrímsson et al., 2004). Similarly, fish lacking one of the MITF orthologues, *mitfa*, have no melanocytes, and ectopic expression of *mitfa* can produce ectopic melanocytes (Lister et al., 1999). Furthermore, the expression of MITF in Medaka embryo-derived stem cells induces differentiation into melanocytes (Béjar et al., 2003), and MITF expression in NIH3T3 cells can activate melanocyte markers (Tachibana et al., 1996). Numerous MITF transcriptional targets have been identified, and include genes encoding components of melanocyte-specific organelles (melanosomes) and the melanin synthesis pathway (see Box 1) as well as more widely expressed genes such as the survival gene *Bcl2* (Cheli et al., 2010). In humans, germline mutations in *MITF* can lead to Waardenburg syndrome or Tietz syndrome [Online Mendelian Inheritance in Man database (OMIM) entries 193510 and 103500, respectively], which are characterised by lack of pigmentation and deafness, as melanocytes also play an important function in the ear. In addition, more subtle changes in MITF transcriptional activity regulated by IRF1 are partially responsible for the pale skin, blue eyes and freckling with brown hair seen in some Northern European populations (Praetorius et al., 2013).

The key step during melanocyte formation is thus the activation of MITF. But how is this achieved? Gene expression studies show that WNT3A is upregulated and bone morphogenetic protein 4 (BMP4) downregulated coincident with melanoblast specification in the quail neural crest, and that WNT3A induces melanoblast formation in quail neural crest cultures (Jin et al., 2001). Furthermore, the addition of WNT3A to mouse melanocytes in culture induces the expression of MITF (Takeda et al., 2000). Clues to the transcriptional mechanism by which *MITF* is activated came initially from mouse and human genetics. Mutations in either of two genes, *PAX3* or *SOX10* (OMIM entries 606597 and 602229, respectively), in humans result in Waardenburg syndrome and a reduction in melanocytes, similar to mutations in MITF, and mice mutant in either gene also lack melanocytes (Tachibana et al., 2003). Both genes also affect the enteric nervous system due to a deficit of neural crest-derived cells (Tachibana et al., 2003).

It was further shown that PAX3 and SOX10 act synergistically to activate *MITF* transcription (Potterf et al., 2000; Watanabe et al., 2002; Bondurand et al., 2000). However, mutant phenotypes and gene expression studies indicate that these transcription factors also specify glial cells, so how are the glial and melanocyte lineages differentiated? Studies suggest that two further transcription factors, FOXD3 and SOX2, play pivotal roles. FOXD3 is expressed in the migrating neural crest cells that give rise to glia and neurons, but is not seen in the later migrating, melanocyte-fated crest cells. The knockdown of *FOXD3* expression in avian neural crest in culture or *in vivo* results in an increase in cells following the melanocyte lineage, whilst *FOXD3* overexpression *in vivo* suppresses melanocyte formation (Kos et al., 2001). In addition, the conditional knockout of *Foxd3* in mice, using *Wnt1-Cre* to ablate the gene specifically in the neural crest, results in MITF-positive cells that are located along developing nerves in the embryo, suggesting that glial-fated cells have been switched to the melanocyte lineage (Nitzan et al., 2013a). FOXD3 acts in the same way in the later differentiating melanoblasts of chick embryos, which share a Schwann cell lineage (Nitzan et al., 2013b). Exploring the mechanism of this switch, Thomas and Erickson (2009) demonstrated that FOXD3, in mouse melanoma cells or in quail neural crest cells, repressed *MITF* expression even in the presence of PAX3 and SOX10. This repression appears to act via a highly conserved motif in the melanocyte-specific promoter of *MITF*, which contains PAX3 and SOX10 binding sites. Curiously, however, chick FOXD3 does not bind to this element, at least *in vitro*; rather, its repressive activity comes about by a mechanism that is independent of DNA binding. FOXD3 interacts with PAX3 and prevents the latter binding to and activating the *MITF* promoter. Indeed, a modified FOXD3 protein, containing a transcription activation domain rather than a repressor domain, still repressed *MITF* transcription in melanoma cells. However, in contrast to these conclusions, repression of zebrafish *mitfa* transcription by FOXD3 is direct; there are FOXD3 binding sites in the zebrafish *mitfa* promoter through which repression is mediated, and the DNA-binding domain of zebrafish FOXD3 is necessary for the repression of transcription in transfected mouse melanoma cells or in fibroblasts co-transfected with SOX10 (Curran et al., 2010, 2009). Very similar results are seen when SOX2 is upregulated or knocked out in chick or mouse embryos. SOX2 binds to the promoter of *MITF* and represses its expression *in vitro*. In line with this, the conditional deletion of *Sox2* in mouse neural crest cells results in a glia-to-melanoblast fate switch, whereas the overexpression of *SOX2* in chick embryos leads to a loss of MITF-positive cells (Adameyko et al., 2012).

The trigger for the melanoblast lineage therefore appears to be downregulation of FOXD3 and/or SOX2 in the glia/melanocyte common progenitor. How exactly this is initiated is unclear, although histone deacetylase 1 seems to be important for repressing *sox10* and *foxd3* expression in the melanocyte lineage in zebrafish (Ignatius et al., 2008; Greenhill et al., 2011). It is also known that the expression of *FOXD3* in the neural crest initially seems to be triggered by ZIC1 and PAX3 (Plouhinec et al., 2014), but is later maintained by SNAIL2 and SOX9 (Nitzan et al., 2013a). There thus appears to be a transcriptional hierarchy in which SNAIL2 activates *SOX9*, which in turn activates *FOXD3*. Thus, persistent expression of *SNAIL2* in chick embryos results in the persistence of *SOX9* and *FOXD3* expression, and the persistence of *SOX9* also gives continuous expression of *FOXD3*, but not *SNAIL2*. Consistently, the expression of dominant negative forms of either SNAIL2 or SOX9 leads to a downregulation of *FOXD3* (Nitzan et al., 2013a). In addition, ectopic expression of MITF in glial-fated neural crest cells in chick embryos downregulates *SNAIL2*, *SOX9* and *FOXD3*, suggesting a mechanism by which, once *MITF* has been activated, there is feedback to reinforce the stability of the melanocyte fate that it programmes. A similar cross-repression mechanism also applies to *SOX2*; ectopic expression of MITF, induced by doxycycline in chick embryos, leads to rapid downregulation of *SOX2* and reinforces the melanoblast fate induced by MITF (Adameyko et al., 2012).

## Melanoblast development and migration in mouse and chick embryos

In order to colonise the epidermis and to completely populate the developing hair follicle field, specified melanoblasts must proliferate to increase their numbers and migrate over large distances. At embryonic day (E) 10.5 in mice, there are fewer than 100 melanoblasts in the trunk region, but by E15.5 this has increased to around 20,000 (Luciani et al., 2011). In organotypic cultures, E14.5 mouse melanoblasts proliferate and appear to migrate randomly at speeds in the order of 0.5 µm min^−1^ (Mort et al., 2010). Failure to migrate or proliferate sufficiently results in the unpigmented patches seen in humans (Fleischman et al., 1991), numerous mouse mutants (Lamoreux et al., 2010) and also in the selectively bred piebald patterns of domestic animals, such as horses and cows (Hauswirth et al., 2012; Fontanesi et al., 2010). As we discuss below, recent studies have begun to identify various populations of melanoblasts and the factors and mechanisms that regulate their migration.

### The dorsolaterally migrating melanoblast population

**Fig. 2.**
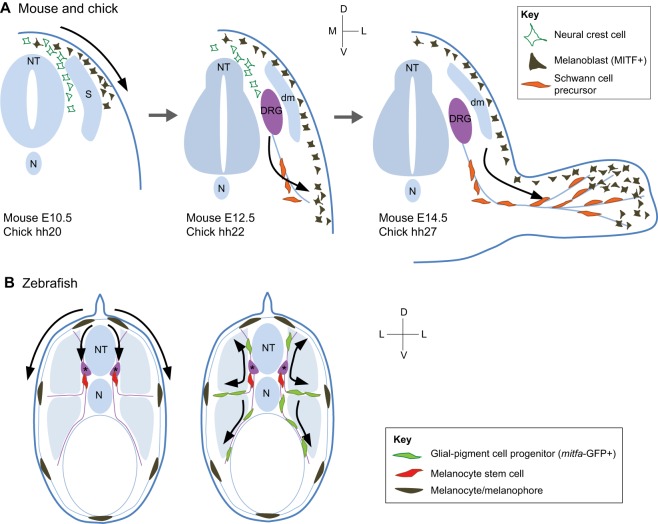
**Development of the melanocyte lineage.** (A) In mouse and chick embryos, early melanoblasts are derived from the neural crest and migrate dorsolaterally through the dermis between the somites and the developing epidermis. At later stages, they become epidermal, and a second wave is thought to differentiate from Schwann cell precursors associated with developing nerves, contributing to the adult melanocytes of the trunk, head and developing limbs. It is not clear whether the late population intercalates with or replaces the early population. (B) In zebrafish embryos, melanocytes migrate dorsolaterally and along nerves ventrally (purple) to form the embryonic pattern. Melanocyte stem cells (MSCs) are located at the DRG (marked by asterisk). Following metamorphosis or melanocyte ablation of the embryonic pattern, zebrafish glial-pigment cell progenitors proliferate and migrate along nerves to form the adult melanocyte stripes. MSCs specified in the developing embryo are the proposed source of metamorphic melanocytes of the adult. Adapted from Adameyko et al. (2009); Dooley et al. (2013); Dupin and Sommer (2012). NT, neural tube; N, notochord; dm, dermamyotome; DRG, dorsal root ganglia; S, somite; D, dorsal; V, ventral; L, lateral; M, medial.

Melanoblast precursors delaminate at around E9 in mouse embryos and begin to express the specific markers *Mitf*, *Dct* and *Pmel*, and from E10.5 (Baxter and Pavan, 2003; Nakayama et al., 1998; Mackenzie et al., 1997) they begin to migrate dorsolaterally through the developing embryo (Fig. 2A). In chick embryos, neural crest migration in the trunk begins at Hamburger–Hamilton stage (HH) 12-13 (Loring and Erickson, 1987). Although ventral migration begins immediately, melanoblasts appear to pause in a region between the somites and the neural tube termed the migration staging area (MSA) before embarking on the dorsolateral pathway from stage 20 (Erickson et al., 1992; Weston, 1991; Wehrle-Haller and Weston, 1995). This dorsolateral migration can be visualised in chimaeras between pigmented and unpigmented mouse embryos, and in retrovirally rescued tyrosinase-expressing mosaic mice, which exhibit broad bands of colour extending dorsolaterally in the adult coat (Huszar et al., 1991; McLaren and Bowman, 1969; Mintz, 1967). The stripes exhibit sharp mid-dorsal separation, indicating that the two sides of the embryo are specified separately. These patterns have been thought to represent clones of migrating melanoblasts that originate from a small number of precursors, but there is a surprising amount of mixing at the axial level between adjacent melanoblast clones, making a prediction of the precise number of progenitors problematic (Wilkie et al., 2002).

Although not required for the initial specification of melanoblasts, endothelin receptor type B (EDNRB; a G-protein-coupled receptor expressed by melanoblasts) and its ligand endothelin B are required for melanoblast migration and proliferation in the dermis between E10.5 and E12.5 (Lee et al., 2003; Shin et al., 1999). If *EDNRB2* (one of the two chick *EDNRB* orthologues) is mis-expressed in early chick neural crest cells, they shift their migration path from ventral to dorsolateral (Krispin et al., 2010), although in the mouse, *Ednrb* is expressed in neural crest cells on both dorsal and ventral pathways.

Changes in the expression of cell adhesion molecules are also required for migration. For example, pre-migratory neural crest populations express the calcium-dependent adherens junction protein N-cadherin, but this is downregulated upon upregulation of *SLUG* (in chicken) or *Snail* (in mice) during the epithelial-to-mesenchymal transition (Nieto et al., 1994; Jiang et al., 1998). Furthermore, at E11.5 in mice, most dermal melanoblasts are E- and P-cadherin negative but, over the next 48 h, there is a 200-fold increase in cadherin expression such that the majority of melanoblasts become E-cad high/P-cad low, concomitant with their migration from the dermis to the epidermis (from E12.5 in mice, HH22 in chick) (Nishimura et al., 1999). It should be noted that crossing from the dermis to the epidermis is an active process but does not require degradation of the basement membrane (Li et al., 2011). By E13.5 the majority of melanoblasts are epidermal and continue to migrate and proliferate in an EDNRB-independent manner. However, not all melanoblasts cross the dermal/epidermal junction and a dermal population also persists. This process is clearly regulated and, although dermal melanoblasts are proliferating, their numbers remain unchanged, indicating a constant and controlled dermal-to-epidermal movement (Luciani et al., 2011). Furthermore, in mice with mutations that cause an expansion of the early melanoblast population, the number of melanocytes reaching the epidermis appears to be unchanged whilst the number of dermal melanocytes increases, thereby resulting in dark skin (Van Raamsdonk et al., 2004), suggesting a mechanism for controlling the size of the epidermal melanoblast population.

The receptor tyrosine kinase KIT and its ligand (KITL) are required for the survival, migration and proliferation of embryonic melanoblasts throughout their migration along the dorsolateral pathway (Yoshida et al., 1996). Mutations in the mouse genes encoding this receptor/ligand pair, which lie within the classical dominant white spotting (*W*) and steel (*Sl*) loci, can result in an absence of melanocytes as well as other developmental defects. KITL occurs in two forms: a secreted, soluble protein and a membrane-bound form found on the surface of keratinocytes in the developing mouse epidermis. In mice, the membrane-bound ligand is absolutely required by migrating dermal and epidermal melanoblasts, regulating their survival and adhesion to the intraepithelial niche (Tabone-Eglinger et al., 2012). Neither form of KITL is required for the specification of melanoblasts or to initiate migration onto the dorsolateral pathway. Dorsolateral entry is, however, preceded by the transient expression of KITL in dermatomal epithelial cells (Wehrle-Haller and Weston, 1995). Signalling through KIT has also been shown to facilitate melanoblast migration and survival through independent mechanisms; migration does not require RAS activity downstream of KIT, whereas survival does (Wehrle-Haller et al., 2001).

### A ventrally migrating population of melanocytes

As discussed above, melanocytes and glia can be derived from a common bipotent neural crest precursor. However, recent evidence suggests that a subpopulation of adult melanocytes in the trunk originates from precursor cells that have Schwann cell/melanoblast bipotential and have migrated on the ventrolateral pathway down the developing nerve sheath. For example, Adameyko et al. (2009) show that, in both chick and mouse embryos, there is a loss of MITF/SOX10-positive melanoblasts from the dorsolateral pathway between E10.5/HH24 and E11.5/HH27, and that these cells are replaced by a second wave of neural crest-derived MITF/SOX10-positive melanoblasts that arise in close proximity to the distal, ventral ramus of the spinal nerve at E12/HH22. If the chick ventrolateral pathway is physically ablated, this late differentiating population is lost, but if the dorsolateral pathway is ablated, it is not. Others have also demonstrated this ventrally migrating melanoblast population in chick embryos (Nitzan et al., 2013b), in which there is more spatial segregation; the dorsally migrating melanocytes populate the epaxial dermis (which is derived from the somatic mesoderm), whilst the ventrally migrating, later differentiating population colonises the hypaxial dermis (which is derived from the lateral plate mesoderm). Whether such a spatial separation of melanocyte progenitors occurs in the mouse is not known; indeed, if and where a distinction between hypaxial and epaxial dermis exists in mice is unknown. In their study, Adameyko and colleagues use *Plp-CreErt2* (Leone et al., 2003) to lineage-mark Schwann cell precursors (SCPs) following tamoxifen injection (Adameyko et al., 2009). Using this approach, they show that a tamoxifen dose at E11 does not mark dorsolateral melanoblasts but does mark the SCP lineage, and that, by P11, 65% of melanocytes in hair follicles can be traced back to these SCPs. It is unclear whether the unlabelled hair follicle melanocytes are derived from SCPs as well but were not efficiently labelled by tamoxifen, or whether they are derived from dorsolaterally migrating cells.

The situation in the head is more complicated, and studies of *Dct-lacZ*-labelled rostrally migrating E10.5 melanoblasts (Mackenzie et al., 1997) and *Pmel17*-labelled laterally migrating E9.5 melanoblasts (Baxter and Pavan, 2003) suggest that several overlapping cranial populations of melanoblasts are probably present. In line with this, Adameyko and colleagues demonstrate (again using *Plp-CreERT2*) a lineage relationship between SCPs and early migrating cranial melanoblasts, but also describe a late migrating population that is not associated with nerves or related by lineage to SCPs (Adameyko et al., 2012). However, it should be noted that the use of *Plp-CreERT2* as an SCP-specific lineage tracer must be evaluated carefully, as it is the key tool in the mouse experiments. Transcriptomic analyses of mouse melanoblasts at E15.5 show that they also express PLP (Colombo et al., 2012), whereas Hari and co-workers showed that *Plp-CreERT2* is active in both nerves and melanoblasts at embryonic stages E11.5, E12.5 and E14.5 (Hari et al., 2012). They further show that Desert hedgehog Cre (*Dhh-Cre*) (Jaegle et al., 2003), another proposed SCP marker, does not label melanoblasts or future melanocytes in the mouse. Furthermore, the transplantation studies of Rawles (1940, 1947) and Mayer (1973) suggest that functional melanoblast populations, able to colonise graft and host tissue, are present in the dermis at E11 and in the dermis and epidermis at E12, overlapping the window in which Adameyko et al. suggest melanoblasts in the dorsolateral aspect become absent (Adameyko et al., 2009). Thus, whilst the common embryonic origin of melanocytes and glia, and the plasticity of the adult Schwann cell, are not in doubt, further work, using alternative approaches to *Plp-CreERT2*, is required to dissect the developmental timings and lineage relationships of melanoblasts and SCPs in the developing mouse embryo.

### Cellular and molecular mechanisms of melanoblast migration

Recent advances in imaging techniques have allowed the observation of melanoblast behaviour in the developing skin using live confocal imaging (Mort et al., 2010, 2014). These approaches have furthered our understanding of melanoblast behaviour at the single-cell and collective level and have provided insights into the regulation of migration and proliferation. Notably, these studies have focused on key players in cytoskeletal organisation and cell migration, such as RAC1, Fascin1, Lamellipodin and the Scar/WAVE complex (Li et al., 2011; Law et al., 2013; Ma et al., 2013).

RAC1 is the main mammalian isoform of RAC and controls actin cytoskeletal assembly mediated by Scar/WAVE and ARP2/3 to drive actin nucleation, and by PAK and LIM kinases to drive the turnover of actin (Delorme et al., 2007; Insall and Machesky, 2009). Melanoblast-specific ablation of *Rac1* in mice leads to migratory defects and problems with cell cycle progression and cytokinesis, resulting in unpigmented patches at both dorsal and ventral positions (Li et al., 2011). Live imaging of melanoblast migration in *Rac1* mutants reveals that melanoblasts mediate their migration using long and short protrusions called pseudopods and stubs, respectively. RAC1 regulates the frequency of pseudopod assembly (Li et al., 2011). Similarly, ablation of the Rac-specific Rho GTPase GEF *Prex1* results in a belly spotting phenotype (Lindsay et al., 2011). Neither *Prex1* nor *Rac1* mutant phenotypes can be rescued by a constitutively active N-RAS, suggesting that proliferation alone is not driving the phenotype (Lindsay et al., 2011; Li et al., 2012). The Scar/WAVE complex is an effector of RAC1 signalling and, in line with this, studies show that the Scar/WAVE complex, in association with lamellipodin (Lpd), regulates melanoblast migration in mice (Law et al., 2013). Melanoblast-specific ablation of Fascin1, a scaffolding protein that is involved in the bundling of actin filaments, is found in both filopodia and invadopodia (Li et al., 2010a; Schoumacher et al., 2010), and is often found in migrating embryonic cells; it also results in a mild belly spotting phenotype and hypopigmentation at the tip of the tail and in the feet (Ma et al., 2013). In these mutants, a delay in cell cycle progression and a reduction in melanoblast number as well as a reduction in pseudopod generation rate (and therefore motility) results in a failure of melanoblasts to fully populate the dorso-ventral axis (Ma et al., 2013).

Surprisingly, the phenotypes in melanoblast-specific knockouts of these genes – *Rac1*, *Lpd* and *Fascin1* – are relatively mild (Li et al., 2011; Law et al., 2013). This implies that melanoblast epidermal migration is probably not strictly an invasive process, as it does not absolutely require these cytoskeletal/motility factors or extracellular matrix degradation activity through matrix metalloproteases (Li et al., 2011; Ma et al., 2013). Work is also required to link the chemokinetic effect of Kit-ligand and its downstream activation of RAS/MAPK and AKT with the cell biology of melanoblast proliferation and migration through regulation of the cytoskeleton.

### Post-migratory behaviour

In mice, the colonisation of the developing epidermis by melanoblasts is complete by E15.5, by which point the primary hair follicle pattern is also established and the secondary pattern is beginning to form (Mann, 1962). From E15.5 onwards melanoblasts begin to colonise the developing hair follicles, and pigment production (see Box 1) then begins at E16.5 (Hirobe, 1984). Little is known about the molecular pathways that control hair follicle localisation but the chemokine SDF-1/CXCL12 has been shown to act as a chemoattractant for melanoblasts through its receptor CXCR4 (Belmadani et al., 2009), whilst soluble KITL has been shown to act as a chemokinetic factor (Jordan and Jackson, 2000). Moving to follicles is associated with a switch from E-cadherin-high/P-cadherin-low to E-cadherin-negative/P-cadherin-medium-high to reflect the cadherin profile of the surrounding keratinocyte environment (Nishimura et al., 1999), suggesting that cell-extrinsic cues are important in regulating the cadherin expression of melanoblasts and that cadherins play a role in positioning melanoblasts in follicles. In early postnatal mice, a follicular and an inter-follicular melanoblast population exists, but in hairy skin, the expression of KITL by keratinocytes declines rapidly after birth and, consequently, the inter-follicular population of melanoblasts disappears a few days after birth, so that none are present in the adult (Hirobe, 1984). In humans, the interfollicular population persists and transfers melanin to the surrounding keratinocytes, thereby pigmenting the skin.

## Melanocyte development in zebrafish

In zebrafish, melanocytes are one of three pigment cell types – melanocytes, iridophores and xanthophores – that form the famous zebrafish stripes. Zebrafish melanocytes arise from a small number of bipotent melanogenic progenitors that are derived directly from the neural crest without a stem cell intermediate during the first three days of development, and from another population that arises via an MSC intermediate (Budi et al., 2011; Dooley et al., 2013; Hultman et al., 2009; Tryon et al., 2011). Evidence of these populations can be clearly distinguished during the patterning of the embryonic and adult stages, and by genetic mutations, some of which give rise to normal embryonic patterns but deficient adult patterns or vice versa (Parichy, 2003). The first wave of melanocytes (referred to as embryonic melanocytes) arises directly from the neural crest and patterns the zebrafish during its embryonic stages by giving rise to four stripes: the dorsal larval stripe, lateral larval stripe, ventral larval stripe and yolk sac stripe (Fig. 2B). These melanocytes traverse a dorsolateral migration pathway between the somites and epidermis to contribute to the larval stripes, as well as a ventromedial pathway, whereby melanoblasts traverse along nerves to contribute to the lateral, ventral and yolk sac stripes (Dooley et al., 2013). As in mammalian melanocytes, KIT functions in the this first wave of melanocytes through distinct pathways, first promoting migration during the first two days of development via its extracellular Ig domains, followed by survival signalling via its cytoplasmic tyrosine kinase domains (Rawls and Johnson, 2003).

Another set of melanocytes then develops in the later stages of larval development and during the transition to an adult zebrafish body shape (metamorphosis) to generate the adult stripes (Hultman and Johnson, 2010). An important series of assays to study these metamorphic or MSC-derived melanocytes in the embryo and adult stages were developed by the Johnson lab, and included the identification of melanocytotoxic small molecules to selectively ablate differentiated melanocytes (Hultman et al., 2009; Rawls and Johnson, 2000; Yang et al., 2004; Yang and Johnson, 2006; O'Reilly-Pol and Johnson, 2008). These studies allow us to infer that melanocyte regeneration following ablation in the embryo occurs from an MSC, the establishment of which is dependent on ERB and KIT signalling (Hultman et al., 2009; Yang and Johnson, 2006; O'Reilly-Pol and Johnson, 2013). Crucially, both embryos treated with an ERB inhibitor in the first few days of development and *erbb3* mutants are defective for both the adult and regenerative melanocytes, suggesting that the MSC population used during regeneration is the same population of cells that generates the adult stripe melanocytes (Budi et al., 2011; Hultman et al., 2009; Hultman and Johnson, 2010). In the adult fin, melanocytes arise from stem cells located at the base of the fin (Tu and Johnson, 2010).

Given the evidence supporting a population of MSCs in the adult zebrafish, the Parichy laboratory undertook studies to successfully identify cells in the adult zebrafish that express neural crest and melanocyte markers (Budi et al., 2011). These extra-hypodermal cells express the *mitfa-GFP* transgenic marker, are dependent on ERB signalling and directly contribute to the adult melanocyte pattern (Budi et al., 2011). Interestingly, these *mitfa-GFP* positive cells are associated with peripheral nerves and ganglia, including the dorsal root ganglia (DRG), ventral motor root fibres lateral line nerves and nerves traversing through the myotome.

An advantage of the zebrafish system is its accessibility to whole-embryo live imaging. To identify MSCs in the zebrafish embryo, Dooley and colleagues from the Nüsslein-Volhard laboratory performed whole-embryo live imaging to demonstrate that a small number of melanoblasts maintain *mitfa*-GFP transgene expression at three days of development, at a time when *mitfa*-GFP transgene expression is normally reduced in differentiated melanocytes, and that these cells contribute to the adult stripes (Dooley et al., 2013). These cells are round and are closely associated with the DRG. *Mitfa*-GFP-positive cells are also associated with the ventral spinal nerves, and the authors note that their elongated shape is suggestive of Schwann cells (Dooley et al., 2013). However, it should be noted that MITF activity itself is not required for MSC establishment because loss of MITFa activity during embryogenesis (by morpholino or a *mitfa* temperature-sensitive mutant allele) still allows full recovery of larval and adult stripe patterns (Dooley et al., 2013; Johnson et al., 2011). The DRG-associated cells are stationary, but, if MITF activity is restored, these cells can proliferate and generate chains of melanoblasts that travel along the peripheral nerves and migrate into the skin (Dooley et al., 2013). Dooley and colleagues thus propose that the DRG is the MSC niche, and that KIT signalling is crucial to recruit and maintain these cells within the niche. The effects of ablating these MSCs are long lasting, and treatment with an ERB inhibitor during the first few days of development in the embryo prevents the future development of adult melanocytes (Budi et al., 2011; Dooley et al., 2013; Hultman et al., 2009). Recent lineage-tracing and live-imaging studies from the Nüsslein-Volhard laboratory indicate that iridophores and melanophores originate from a shared niche at the DRG, and that, having followed the tracts of peripheral nerves to the skin, the melanoblasts then migrate in the skin until they reach the stripe, differentiate into melanocytes and grow dramatically in size (Singh et al., 2014).

Little is known about the regulation of the dramatic pigmentation changes that occur during zebrafish metamorphosis. Given the role of thyroid hormone (TH) in metabolism and growth, the Parichy laboratory hypothesised that adult pigment patterns might be under hormonal control (McMenamin et al., 2014). They discovered that a genetic activating mutation in *thyroid stimulating hormone receptor* (*tshr*) leads to a loss of melanocytes and an increase in yellow xanthophores, whereas targeted genetic ablation of the thyroid, or genetic loss of function mutation in *tshr*, leads to an increase in melanocytes and loss of xanthophores. Notably, ablation of TH in adult fish with established stripes lead to an increase in melanocyte numbers, indicating that TH plays an active role in repressing homeostatic melanocyte numbers. This might be relevant for melanoma because hypothyroidism patients are at increased risk for melanoma (Shah et al., 2006).

## Mammalian MSCs

**Fig. 3.**
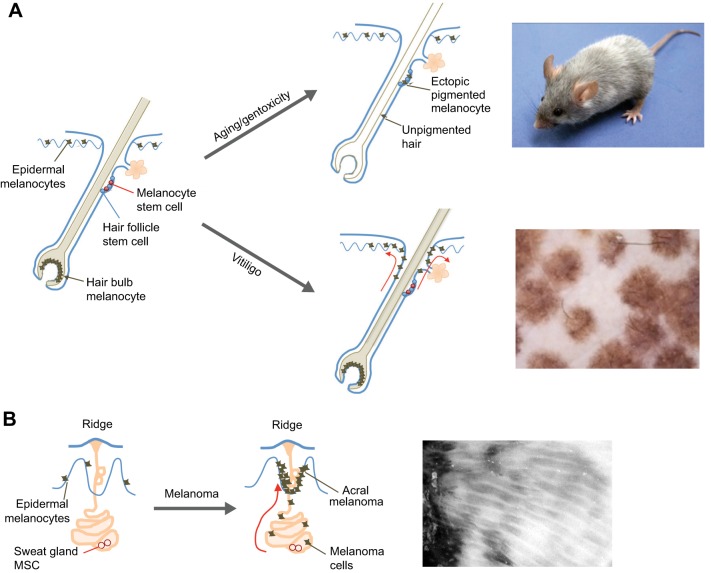
**Melanocyte stem cells in ageing and disease.** (A) Melanocyte stem cells (MSCs; pink circles) associated with the hair follicle provide pigmented cells to the growing hair. These follicular MSCs reside in the bulge region of the hair follicle and are supported in a niche by hair follicle stem cells (blue). Differentiated melanocytes (black) reside at the bulb to pigment the growing hair. Aging or genotoxicity lead to the ectopic differentiation of MSCs in the follicular niche, resulting in a loss of the MSC pool and hence loss of pigmentation of the hair. This gives rise to grey hair, as illustrated in the image of a mouse (far right), in which genotoxic stress caused by ionizing radiation has induced hair greying [image courtesy of Emi Nishimura (Tokyo Medical and Dental University, Tokyo, Japan)]. The follicular MSCs also act as a reservoir for epidermal melanocytes in vitiligo patients, such that melanocytes from the MSC population migrate (indicated by red arrows) to the skin. This results in the patches of pigmented skin associated with hair follicles that are observed in vitiligo patients, as illustrated in the image on the right. [Image from Grichnik (2008) with permission.] (B) MSCs (pink circles) have also been identified in the sweat glands of volar skin and can contribute to the epidermal melanocyte population (black). The sweat gland can also provide a niche for melanoma-initiating cells (black), which explains the ‘parallel ridge pattern’ of acral melanoma cells at the sweat gland [illustrated in the image on the far right; image from Saida et al. (2002) with permission]. Schematic images adapted from Nishimura (2011); Zabierowski et al. (2011).

Returning to mammalian melanocytes, once melanocyte development and migration are established, how do animals maintain, alter or regenerate pigmentation in adult life? Evidence from multiple species points to a reservoir of MSCs that can be called upon when needed to re-pigment the skin and/or hair (Fig. 3). The identification and potential manipulation of MSCs might hold therapeutic value for pigmentation disorders, wounding and melanoma.

### Melanocyte reservoirs in the skin and hair

The concept of a reservoir of undifferentiated melanocytes that becomes activated to replenish depleted melanocytes comes from initial observations in vitiligo patients (Nishimura, 2011). Vitiligo is a condition that is characterised by a loss of melanocytes and associated pigmentation in the skin and hair. In the early stages of vitiligo, narrow-band UVB therapy can stimulate repigmentation to give rise to tiny islands of pigmentation that eventually become joined to form patches of uniform pigmentation (Fig. 3A). These islands have a hair follicle at their centre, suggesting that melanocytes from the follicle are the source of the new skin melanocytes. Indeed, early studies (Nishimura, 2011; Staricco, 1959, 1963) identified a population of cells in the outer root sheath of the lower permanent portion of the hair follicle that lacks melanin (i.e. is amelanotic) and that becomes activated following UV irradiation or epidermal wounding, and hence acts as a reservoir for skin melanocytes (Cui et al., 1991). Whilst evidence for a melanocyte reservoir in the human hair follicle was emerging, a similar reservoir was also becoming apparent in mice. KIT is required for the survival of melanocytes, so administering a mouse with anti-KIT antibody leads to hair greying by inhibiting melanocyte proliferation and differentiation (Botchkareva et al., 2001). Subsequent hairs, however, are pigmented, indicating that a KIT-independent progenitor population resides in the hair follicle (Botchkareva et al., 2001). A crucial finding came from Nishimura and colleagues, who identified this KIT-independent population as the MSCs (Nishimura et al., 2002).

More recent studies show that, during development, and once at the hair follicle, some melanoblasts differentiate to pigment the first hair cycle, whereas others remain associated with the bulge region and hair stem cells, reduce their expression of *Mitf* and become quiescent MSCs until activated in the following hair cycle (Nishimura, 2011). These quiescent MSCs reside in the lower permanent portion of the hair follicle as undifferentiated, slow-cycling melanoblasts (Fig. 3A), marked by expression of a *Dct:lacZ* transgene, and provide the hair matrix with amplifying and differentiating melanocytes for that hair unit. There are no markers unique to these MSCs, but they are characterised by their cellular shape and location within the hair follicle, their low proliferative rate and their low levels of KIT and MITF expression (Nishimura et al., 2002; Ueno et al., 2014). Transcriptomic analysis suggests that these cells have very low transcription rates and are in a quiescent state, only to be stimulated to initiate gene expression and cellular activity at the beginning of the hair follicle growth stage (anagen) (Osawa et al., 2005; Freter et al., 2010).

Experimental evidence indicates that the MSCs in mice can function in a similar manner to the amelanotic population in the lower permanent portion of the human hair follicle. Unlike human skin, the hairy skin of the mouse does not contain melanocytes and does not express *Kitl*. However, the ectopic, transgenic expression of *Kitl* by keratinocytes enables the survival of melanocytes in the mouse skin, providing an opportunity to generate mice with melanocytes in the hair and skin (Kunisada et al., 1998), as in humans. As mentioned above, treating neonatal mice with a KIT-blocking antibody selectively eliminates KIT-positive differentiating melanocytes while sparing the MSCs. Therefore, at the next anagen hair follicle stage, the MSCs become activated and generate differentiating, transit-amplifying daughters. These melanocytes pigment the hair and, in transgenic mice expressing KITL in the skin, are also able to migrate, survive and pigment in the skin. Importantly, similar to repigmentation of vitiligo patients, islands of melanocytes repopulate the skin from the hair follicle, providing experimental evidence that the follicular MSCs in the mouse can provide a reservoir for hair and skin melanocytes (Nishimura et al., 2002).

Insights into the pathways that govern MSCs have also been gained from studies of the visible grey hair pigmentation phenotypes associated with defective MSCs. These include the phenotypes that arise following perturbation of the BRAF and CRAF kinases, which regulate cell cycle entry (Valluet et al., 2012), BCL2, which controls survival (Nishimura et al., 2005; Mak et al., 2006), and SOX10, which is required for maintenance within the bulge and for replenishing differentiated melanocytes in the bulb (Harris et al., 2013). Notch signalling is also important for the maintenance of melanoblasts and MSCs, both during development and in the adult (Moriyama et al., 2006; Schouwey et al., 2007; Kumano et al., 2008; Aubin-Houzelstein et al., 2008). Notably, the expression of Notch1 is sufficient to convert pigmented mature primary human melanocytes to multipotent neural crest stem-like cells, underscoring the importance of Notch signalling in melanocyte programming (Zabierowski et al., 2011).

Once at the follicular niche, the transit-amplifying progeny of MSCs can colonise neighbouring empty niches and become MSCs for a new hair unit, or they can revert back to a quiescent MSC state following selective ablation of the stem cell pool, for example by irradiation or genotoxic drugs (Nishimura et al., 2002; Ueno et al., 2014). In the niche, TGFβ plays a crucial role in regulating the transition of MSCs into a quiescent state during the hair cycle, acting by inhibiting differentiation (pigmentation) gene expression and by initiating BCL2-dependent cell cycle arrest (Nishimura et al., 2010). Within the bulge region, the MSCs intimately interact with hair follicle stem cells (HFSCs) that are anchored by collagen XVII and provide a functional MSC niche (Fig. 3A). The two stem cell populations coordinate hair growth with pigmentation via TGFβ and WNT signalling cross-talk (Tanimura et al., 2011; Rabbani et al., 2011). Recent evidence also implicates the transcription factor NFIB as a coordinator of this cross-talk; inhibition of endothelin 2 by NFIB in HFSCs prevents enhanced proliferation and precocious differentiation in the MSCs (Chang et al., 2013).

Although much of our understanding about MSCs comes from the hair follicle-associated MSCs, two additional sources of melanocytes that are important for the identification of MSCs in the skin are emerging. The Herlyn laboratory developed three-dimensional (3D) reconstruction skin models from human foreskin, which lacks hair follicles, and discovered that, when dermal stem cells are embedded into dermis, a subpopulation migrates to the basement membrane of the epidermis and differentiates into melanocytes (Li et al., 2010b). Thus, dermal stem cells might have the potential to replenish extra-follicular epidermal melanocytes. Another source of melanocytes appears to be in the sweat glands of acral volar skin in humans and mice (Fig. 3B); the Nishimura laboratory recently identified the secretory lower permanent portion of the sweat gland as a new anatomical niche for MSCs that can provide the epidermis with differentiated melanocytes (Okamoto et al., 2014). Crucially, this niche can also maintain melanoma cells in an immature state, which thus explains the characteristic dermoscopic ‘parallel ridge pattern’ formed by acral melanoma cells (Okamoto et al., 2014) (Fig. 3B).

### MSC depletion in aging, disease and wounding

Despite their potential for renewal, MSCs are a limited population. A gradual decline in the number of follicular MSCs during physiological aging results in hair greying in both humans and mice (Nishimura et al., 2005). MSCs in zebrafish also have the potential for repeated renewal after sequential ablation, and, although zebrafish do not ‘go grey’, these MSCs can eventually become depleted, resulting in loss of hypodermal melanocyte stripes (Budi et al., 2011).

Although little is known about MSC depletion in zebrafish or chick, work from Nishimura and colleagues demonstrate that the physiological depletion of MSCs in the niche is accompanied by cellular differentiation (Nishimura et al., 2005). In older mice and humans, ectopically pigmented (differentiated) melanocytes (EPM) become apparent at the bulge region of the outer root sheath and are associated with the loss of MSCs (Nishimura et al., 2005). MSC depletion is also observed in hair greying induced by DNA damage, such as that induced by X-rays or chemotherapeutic drugs, but the response is distinct from the DNA damage pathway that triggers apoptosis or senescence of other stem cell populations (Inomata et al., 2009). Interestingly, this DNA damage pathway is p53 independent and instead relies on an Ataxia-telangiectasia mutated (ATM) MSC ‘stemness checkpoint’ (Inomata et al., 2009). The appearance of EPMs following DNA damage suggests that EPMs seen in aging individuals are caused by an accumulation of DNA damage, and supports the idea that one of the major causes of stem cell depletion is the accumulation of DNA damage over one's lifetime.

What about premature hair greying? In fact, individuals with mutations in the DNA damage repair pathways, such as those affected by premature aging syndrome, Werner's syndrome (caused by mutation in the ReqQ helicase WRN) or Ataxia-telangiectasia (caused by mutation in ATM), do exhibit premature hair greying (see Box 2), indicating that the DNA damage response is important for maintaining stem cell renewal. Premature hair greying, without aging in other systems, is observed in patients carrying MITF mutations and in the mouse *Mitf^vitiligo^* mutation. These examples from human and mouse genetics indicate that, despite being expressed at low levels in the MSC, MITF is important for maintaining the stem cell pool. It is tempting to speculate that this might be linked to the DNA damage response, because EPMs are observed following DNA damage induced by prolonged expression of the melanogenic genes regulated by MITF (Inomata et al., 2009), and because MITF directly regulates DNA damage repair genes in melanoma (Giuliano et al., 2010; Strub et al., 2011). It will also be interesting to determine whether EPMs are able to play a role in preventing melanoma by removing damaged stem cells.

Box 2. **Premature greying syndromes**

Hair pigmentation is one of the most striking phenotypes in humans. Most humans begin to show grey hair at about 35 years of age, but some people develop grey hair prematurely – before 20 years in Europeans, 25 years in Asians and 30 years in Africans (McDonough and Schwartz, 2012; Pandhi and Khanna, 2013). The aetiology of premature hair greying is not always known, but it can be symptomatic of underlying autoimmune disorders such as vitiligo, or endocrine disorders such as hypothyroidism (McDonough and Schwartz, 2012; Pandhi and Khanna, 2013). Genetic conditions affecting melanocyte biology, including Waardenburg Type II syndrome and Tietz syndrome (caused by mutations in MITF and SOX10), are also characterised in part by premature hair greying and, as with physiological hair greying, the greying is associated with a loss of melanocytes (Tassabehji et al., 1995). Premature hair greying also features in segmental progeroid syndromes, rare genetic syndromes of accelerated aging of multiple organs and tissues, including dyskeratosis congenital (which is caused by mutations in TERT), Hutchinson–Gilford progeria syndrome (which involves mutations in Lamin A/C) or syndromes caused by defective DNA repair, such as Werner syndrome, Cockayne syndrome or xeroderma pigmentosum (Sahin and Depinho, 2010). Environmental factors, such as UV light (Trueb, 2006), smoking (Mosley and Gibbs, 1996) and oxidative stress (Shi et al., 2014; Wood et al., 2009; Kamenisch and Berneburg, 2009) might also contribute to premature hair greying, possibly through an increase in intracellular reactive oxygen species. Nutritional deficiencies in trace elements (Fatemi Naieni et al., 2012), including vitamin D3 (Bhat et al., 2013), vitamin B12 (such as in pernicious anaemia) and copper (such as in Menkes disease) can also lead to hair greying. These conditions are thought to reflect deficiencies in the pigmentation pathway rather than loss of melanocytes, such that colour can be restored with nutritional supplementation (Heath and Sidbury, 2006).

MSCs also have the potential to leave the niche. Recent experiments reveal that MSCs themselves, rather than proliferating daughter cells, migrate out of the hair follicle niche in an MC1R-dependent process to provide a source of melanocytes in mouse skin during wound repair or following UV-irradiation (Chou et al., 2013). Notably, in the context of wound repair, the migration of MSCs from the niche can be so drastic that it empties the hair follicle niche, resulting in white hairs (Chou et al., 2013). However, wound-induced epidermal melanocytes – those that seem to be able to produce pigment in the skin – can be recruited to newly forming hair follicles to take their place as MSCs (Chou et al., 2013). Wounds can also be associated with hyperpigmentation, and this could be because the wound-healing process produces a chemokine that attracts melanocytes (Chou et al., 2013), or it could suggest a non-canonical function for melanocytes in wound repair. In zebrafish, wounding induces hyperpigmentation by recruiting melanoblasts and melanocytes to the wound site, and this is dependent on the activity of macrophages and neutrophils at the wound (Levesque et al., 2013). In mammals, communication between macrophages and melanocytes in response to UV (Zaidi et al., 2011), together with the existence of macrophage-like melanocytes that reside in the perivasculature (Zhang et al., 2012, 2013) and respond to inflammation (Bald et al., 2014), provide additional evidence that melanocytes might have the potential to do more than just colouring skin and hair cells.

## The melanocyte lineage in melanoma

One of the most deadly and metastatic forms of cancer is melanoma – cancer of the melanocyte. Clues to melanoma therapies come from directly understanding melanocyte development. Indeed, there are an increasing number of examples that show correlations between genes that regulate neural crest/melanocyte development and those that contribute to melanoma (Shakhova, 2014; White and Zon, 2008). Understanding the ontogeny and biology of melanocytes – their development from stem and progenitor cells, their proliferation, migration, differentiation, regeneration and death, and their interactions with their environment – might be highly informative for generating new therapeutic approaches to pigmentation disorders and melanoma.

Activating mutations in *BRAF* are among the most frequent mutations in melanoma, and somatic mutations have also been identified in genes encoding the transcriptional machinery that drives neural crest and melanocyte development. For example, a link between MITF and melanoma was first established by histopathology in human melanoma, and many MITF molecular mechanisms and target genes in melanoma have subsequently been revealed (Cheli et al., 2010; Tsao et al., 2012). The expression of MITF can lead to the expression of cell cycle genes that both promote and inhibit melanoma progression, implying a ‘rheostat model’ in which MITF alters expression of different target genes, depending on its expression and activity level. At the most basic level of this idea, high levels of MITF function to promote melanocyte differentiation and cell cycle arrest, whereas lower levels of MITF activity activate the cell cycle without promoting the expression of pigmentation genes (Hoek and Goding, 2010). Interestingly, the *MITF* mutations that are associated with Waardenburg and Tietz syndromes are distinct from those that are found in melanoma: Waardenburg and Tietz syndrome *MITF* mutations primarily alter the DNA-binding properties of MITF and inactivate its transcriptional activity, whereas melanoma *MITF* mutations confer altered transcriptional activity upon MITF (Grill et al., 2013; Cronin et al., 2009). For example, the expression of MITF harbouring the melanoma-associated *MITF^4T^*^Δ*2B*^ mutation in zebrafish does not inhibit the specification or differentiation of melanocytes but instead allows the continued proliferation of differentiated melanocytes during development (Taylor et al., 2011). It was also shown that, in zebrafish, a hypomorphic *mitfa* mutation cooperates with oncogenic BRAF to promote superficial spreading melanoma (Lister et al., 2014). Furthermore, in horses, a mutation in *MITF* causes premature hair greying coupled with melanoma (Curik et al., 2013; Rosengren Pielberg et al., 2008). MITF is expressed in both melanocytes and the kidney, and a germline mutation in human *MITF* that predisposes to melanoma and kidney cancer has recently been found. This *MITF^318K^* mutation leads to altered MITF activity and, although the transcription of some melanogenic genes remains unchanged, one consequence of the mutation is transcription of hypoxia-inducible factor 1-alpha (HIF1-α), which provides a growth advantage to cancer cells (Bertolotto et al., 2011; Ghiorzo et al., 2013; Yokoyama et al., 2011).

Links between SOX10 and melanoma have also been identified and, as such, SOX10 might be an effective target for melanoma and for giant congenital nevi, which are benign melanocyte-containing skin lesions that can sometimes develop into melanoma. Sommer and colleagues noticed similar histopathological features between children with giant congenital nevi and mice that express activated N-RAS in melanocytes: both have nests of pigmented nevus cells in the dermis without apparent epidermal involvement, and both human and mouse nevi melanocytes express higher levels of Sox10 than control melanocytes (Shakhova et al., 2012). Loss of the melanoma tumour suppressor INK4A (CDKN2A – HUGO Gene Nomenclature Committee) leads to rapid melanoma formation in activated N-RAS-expressing mice and, in a remarkable genetic experiment, it was shown that the loss of one allele of *Sox10* is sufficient to counteract activated N-RAS hyperpigmentation and melanoma (Shakhova et al., 2012). Similar findings by the Pavan laboratory demonstrate that *SOX10* haploinsufficiency prevents melanoma formation in a *Grm1*-transgenic mouse melanoma model (Cronin et al., 2013). Understanding the downstream effectors of lineage-specific programmes in melanoma will be crucial. In a recent example, Soengas and colleagues identified RAB7 as a new regulator of melanoma progression and showed this to be specifically wired into the melanoma lineage by the transcription factors SOX10 and MYC (Alonso-Curbelo et al., 2014).

The dependence of melanoma and nevi on melanocyte developmental factors might be an exploitable feature in the clinic. *Sox10* haploinsufficiency in mice can lead to tumour regression in an already established nevus, and in zebrafish, turning off MITF activity in an established melanoma leads to rapid tumour regression (Lister et al., 2014; Shakhova et al., 2012). Furthermore, recent genomic analyses of human melanomas have revealed that MITF and a melanocyte lineage-specific programme are especially important in drug-resistant melanomas, suggesting that MITF is involved in drug-resistance mechanisms (Johannessen et al., 2013).

## Conclusions

In recent years, great progress has been made in understanding the molecular events that lead a neural crest cell to become a melanocyte, how these melanocytes are maintained during homeostasis, and how melanocytes respond to their environment. The interplay between transcription factors resulting in expression of the ‘master regulator’ MITF is being explored, and some knowledge exists of the roles played by SOX10, PAX3, FOXD3, SOX2, SOX9, ZIC3 and SNAIL2. There appear to be mechanistic differences between, for example, chick and zebrafish, and it remains to be seen whether these are real species-dependent features or whether they simply reflect differences in experimental approaches. Some of the extrinsic factors that determine or guide melanocyte differentiation, such as WNT3A, KIT-ligand and EDN3 (the ligand of EDNRB), have also been identified but there might well be more to find. In particular, thyroid-stimulating hormone has been shown to participate in zebrafish pigment cell development but has not, as yet, been directly implicated in melanocyte development in other vertebrates.

The recent findings that melanocytes can arise from neural crest cells on a ventral migratory pathway, which can be identified as Schwann cell progenitors by their gene expression, are provocative. Certainly, melanocytes in zebrafish can arise from precursors migrating along nerves, and in chick, a dual origin, depending on the mesodermal source of the dermis, has been demonstrated. Establishing the degree to which these novel progenitors contribute to functional melanocytes in mice probably needs the development of better lineage-restricted Cre-driver mice.

The biology of MSCs in various species has also been well examined recently. In mice, much of the work has focussed on the readily identifiable stem cells present in the hair follicle and, latterly, those in the sweat glands. It is likely, however, that there are also stem cells in the interfollicular epidermis that remain to be found. Any roles for these stem cells in melanoma formation and/or in vitiligo might point the way to future therapies for these diseases. Overall, understanding the ontogeny and biology of melanocytes will undoubtedly be highly informative for developing new therapeutic approaches to pigmentation disorders and melanoma.
